# The first intron of ARF7 is required for expression in root tips

**DOI:** 10.1016/j.isci.2024.109936

**Published:** 2024-05-08

**Authors:** Jingyi Han, Thomas Welch, Ute Voß, Teva Vernoux, Rahul Bhosale, Anthony Bishopp

**Affiliations:** 1 School of Biosciences, University of Nottingham, Loughborough, UK; 2 Department of Biosciences, Durham University, Durham, UK; 3 Laboratoire Reproduction et Développement des Plantes, Université de Lyon, ENS de Lyon, CNRS, INRAE, Lyon, France; 4 International Crops Research Institute for the Semi-Arid Tropics (ICRISAT), Patancheru 502324, Telangana, India

**Keywords:** Molecular biology, plant biology, Botany

## Abstract

Auxin regulates plant growth and development through the transcription factors of the AUXIN RESPONSE FACTOR (ARF) gene family. ARF7 is one of five activators that bind DNA and elicit downstream transcriptional responses. In roots, ARF7 regulates growth, gravitropism and redundantly with ARF19, lateral root organogenesis. In this study we analyzed *ARF7 cis*-regulation, using different non-coding sequences of the *ARF7* locus to drive GFP. We show that constructs containing the first intron led to increased signal in the root tip. Although bioinformatics analyses predicted several transcription factor binding sites in the first intron, we were unable to significantly alter expression of GFP in the root by mutating these. We instead observed the intronic sequences needed to be present within the transcribed sequences to drive expression in the root meristem. These data support a mechanism by which intron-mediated enhancement regulates the tissue specific expression of *ARF7* in the root meristem.

## Highlights

• The first intron of *ARF7* is required for expression in the root apical meristem
• It is not required for expression in either lateral roots or the shoot apex
• We find no binding sites in the first intron required for expression in root tips
• We propose that this intron exerts its effects via intron-mediated enhancement

## Graphical abstract

**Figure:**
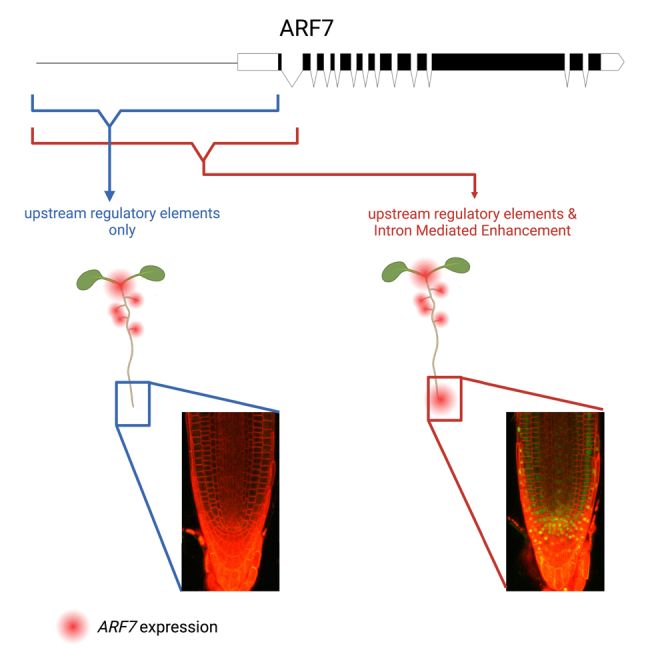

## Introduction

Auxin plays an essential role in regulating growth and developmental processes in plants, such as the formation and maintenance of shoot apical meristem (SAM) and root apical meristem (RAM), vascular patterning, establishment of phyllotaxy, shoot phototropism, root gravitropism, and female gametophyte development.^1^^,^^2^ Several gene families comprise the auxin signaling network and mediate transcription of downstream responses genes based on cellular auxin levels. The binding of auxin promotes the interaction between the SCF^TIR1^ ubiquitin protein ligase complex and the Aux/IAA co-repressors.^3^ The SCF-type complex comprises an F box protein^4^ alongside the Skp1 and Cullin proteins.^5^ This complex transfers an activated ubiquitin from a ubiquitin-activating enzyme and conjugates the Aux/IAAs.^6^^,^^7^ The Aux/IAAs themselves act to stabilize auxin binding, leading to them being considered as co-receptors for auxin.^8^ Following the ubiquitination of the Aux/IAAs, these proteins are degraded by the 26S proteasome.^9^^,^^10^ In addition, TIR1/AFB synthesizes cAMP upon AUX/IAA binding, which is essential for a normal auxin response.^11^ Transcription Factors (TFs) of the AUXIN RESPONSE FACTOR (ARF) family^12^ bind to Auxin response elements within DNA.^13^ The Aux/IAA proteins bind some of these ARFs and inhibit their transcriptional activity.^14^ Under high auxin concentrations, the Aux/IAA proteins are degraded, thus allowing ARFs to regulate thousands of downstream responses.^15^^,^^16^

In Angiosperms, auxin signaling component families are encoded by large gene families. For example, the Arabidopsis genome encodes 23 ARF proteins that control distinct developmental processes.^17^ They are divided to 3 clades based on phylogenetic analysis. Clade A has 5 members, including ARF5, ARF6, ARF7, ARF8, and ARF19^18^ that have been shown to be transcriptional activators^17^ and to be all bound by Aux/IAAs. As activators, they bind in promoter or enhancer regions of target genes to initiate or enhance transcription. Alongside these activators, ARF repressors belonging to the two other clades are thought to bind similar regions to inhibit the expression of auxin target genes.

These five Clade A activator ARFs (A-ARFs) play key specific roles in development of Arabidopsis. To understand how A-ARFs can mediate diverse functions throughout development, the expression of A-ARFs alongside other ARFs has been mapped at the cellular scale.^19^^,^^20^^,^^21^ In both the root and shoot apical meristems as well as in other organs,^17^^,^^20^^,^^22^^,^^23^ each A-ARF shows a unique but partially overlapping expression pattern and it is likely that each ARF or combination of ARFs regulate a different subset of targets. Consistently, over-expression of ARFs causes distinct phenotypes that are not present when other ARFs are overexpressed. For example, overexpression of *ARF5* can restore the *arf7* hypocotyl elongation phenotype, but overexpression of *ARF7* cannot restore the vascular defects in *arf5*.^24^ While there is a certain degree of genetic redundancy within these components, there is evidence that the spatial patterns of ARF response are important for controlling the spatial specificity of auxin responses. Accordingly, either individual ARFs or pairs of ARFs have been associated with specific developmental responses.

ARF7 and ARF19 are well known for their redundant role in regulating lateral root organogenesis via activation of the transcription factors LATERAL ORGAN BOUNDARIES-DOMAIN (LBD)16 and 29^17^^,^^25^^,^.^26^ ARF7 and 19 also control other processes within the root, such as cell wall composition and pectin dynamics during root hair tip growth through ERULUS,^27^ adventitious root formation via regulating LBD16 and LBD18^28^ as well as in tropic responses such as gravitropism.^17^^,^^29^ In the aerial parts of the plant, besides the hypocotyl aforementioned, ARF7 is expressed in the shoot meristem^21^ as well as in veins of maturing leaves, especially in older procambial strands, where it works together with other activating ARFs to control leaf formation.^30^ ARF7 also works redundantly with ARF19 to control expansion of leaf cells.^31^

Recently we investigated the regulatory networks regulating the five A-ARFs and found that at these loci chromatin was generally open and that transcription was predominantly controlled by a collection of transcriptional repressors.^20^ The genomic structure of the five class A ARFs generally featured a large first intron. As intronic sequences had previously been implicated in regulating expression patterns,^32^^,^^33^ the expression of all five activating ARFs was investigated in transgenic reporters containing either only sequence 5′ to the translational start site or in reporters containing an in-frame fusion of GFP to the second exon.^20^ ARF7 showed an interesting expression pattern that deviated to those we observed for the other ARFs. While for ARFs 5, 6, 8, and 19, there was no discernible difference between the two regulatory fragments, there was a clear difference in the ARF7 expression pattern. A broad expression of *ARF7* was observed in root tips only in reporters including DNA sequences downstream of translational start, while both reporters produced similar expression patterns in shoots. This study suggested the presence of regulatory elements either in the first intron or first exons guiding expression in roots but not shoots. Given that these regulatory sequences appeared to increase GFP transcription, we hypothesized that transcriptional activators would likely bind these sequences. However, this was in sharp contrast with the majority of components identified within the A-ARF regulatory networks that were predicted to be transcriptional repressors. In this paper, we explore the role that sequences downstream of the translational start site (including the first intron) play in regulating ARF7 expression and identify an organ-specific role for the first intron in boosting expression of *ARF7* mRNA in the root meristem.

## Results

### The first intron of ARF7 is required to drive expression in the root apical meristem

**Figure 1:**
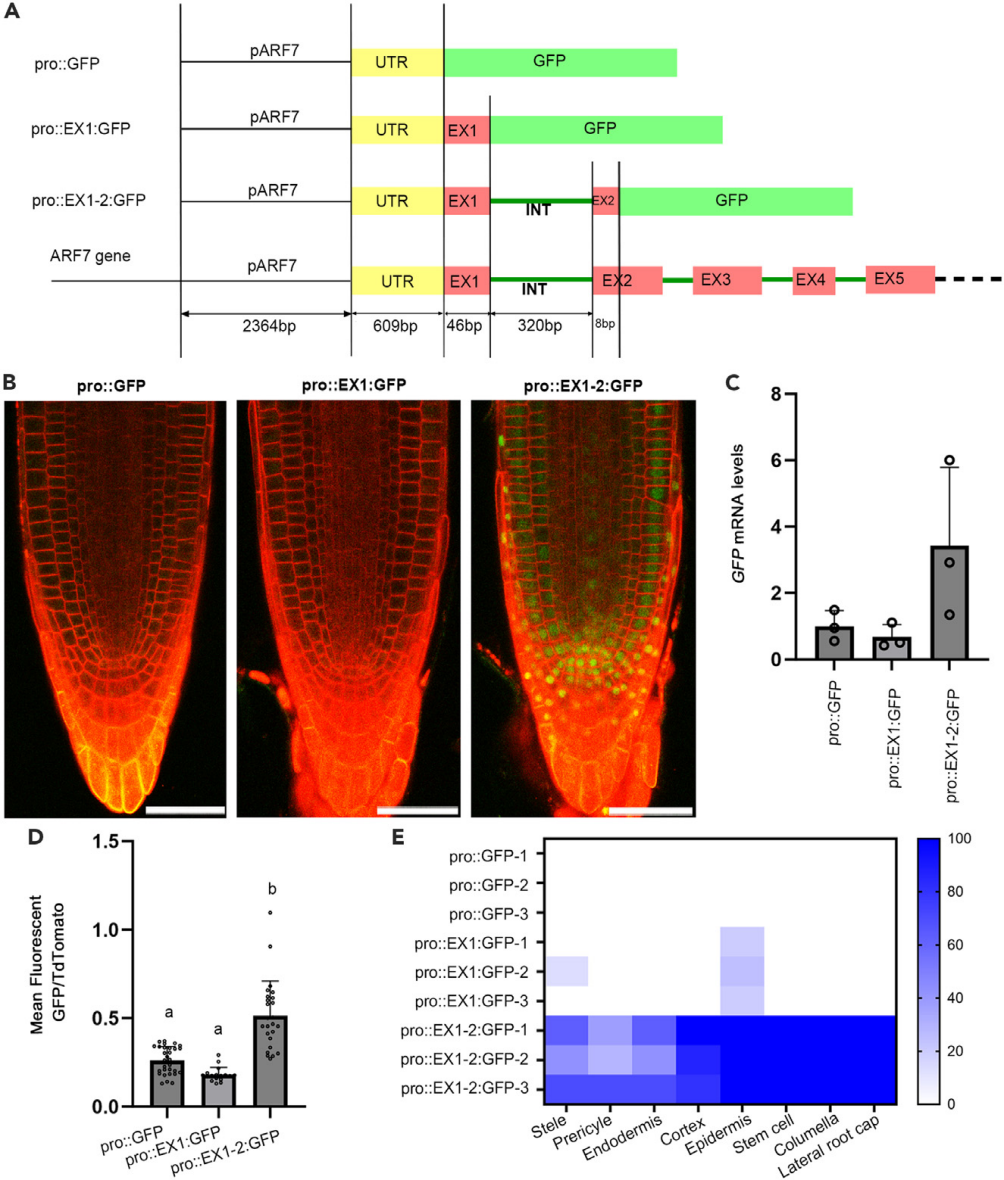
The first intron of ARF7 is required to drive strong expression within the root apical meristem (A) Schematic diagram showing different ARF7 promoter configurations used in this project. Pro, promoter; EX, exon; INT, intron. Please see Supplemental Data for exact plasmid sequences. (B) Confocal images showing expression of GFP (Green) driven by the ARF7 promoter the in RAM. Cell membranes are visualized with td-Tomato using the DEAL system. Scale bars is 50μm. (C) GFP expression level quantified by RT-qPCR in 2mm root tips. Error bars are standard deviation of three independent transgenic lines. (D) ARF7 expression level quantified in the proximal meristem zone by the ratio of mean fluorescent GFP/td-Tomato. All constructs were measured based on 3 independent transgenic lines, for pro:GFP each line included 10, 8, and 12 individuals, for pro:EX1:GFP each line included 5, 5 and 7 individuals, for pro:EX1-2:GFP each line included 8, 10, and 7 individuals. Error bars are standard deviation of samples. Significant test was done by ANOVA with Tukey HSD. *p* < 0.05 (E) Heatmap showed the percentage of roots displaying clear nuclear localized GFP in each cell-type in the RAM. The scale is %. Stem cell refers to the quiescent center and adjoining stem cells.

Our previous work had shown different expression patterns for *ARF7* in the root when driven by a promoter including only sequence 5′ of the translational start site to those when the Venus was present as an in-frame fusion to the second exon.^20^ To test whether these differences were due to the inclusion of the first intron, rather than a result of either binding sites within the first exon or changes in translatability of the protein, we designed a series of constructs to separate these possibilities. We exploited recent advances in greengate cloning^34^ using the dual expression and anatomy lines (DEAL).^35^ Alongside the gene of interest, the DEAL vectors introduce a red plasma membrane marker (TdTomato) under the ubiquitously expressed *UBQ10* promoter, and act as a control showing the activity of the transgene. Initially, we created two constructs, one that contained only 5′ sequence upstream of the translational start site and the other included an in-frame fusion to the second exon (Figure 1A). Similar to previous reports, the construct containing only 5′ sequence (pro:GFP) showed virtually no GFP fluorescence in the RAM, while the construct including the first intron by way of a fusion between the second exon and GFP (pro:EX1-2:GFP) showed broad expression in the RAM, including within the stele, endodermis, cortex, and epidermis (Figures 1B and 1E). Both constructs showed roughly equivalent levels of TdTomato signal.

To test whether sequences in the first exon or first intron were responsible for the altered pattern, we created a new reporter line including an in-frame fusion with the first exon (pro:EX1:GFP). Similarly to reporter constructs driven by the 5′ sequence alone, we saw little expression within the root meristem (Figure 1B). There was some variation in signal between individual plants. GFP signal was observed in the root tips of a few individual plants, however, in these cases the signal was very weak and restricted to the epidermis (Figure 1E). *GFP* mRNA levels were quantified by RT-qPCR and were lower in both constructs without the intron compared to the one containing the intron (Figure 1C). As the coding sequence included within pro:EX1:GFP and pro:EX1-2:GFP constructs differed by only 8bp, these results strongly supported the theory that it is the first intron, rather than first exon, that plays an important role in defining ARF7 expression within the RAM.

To test if the first intron affected ARF7 expression in other parts of the root, we observed different tissues with pro:EX1:GFP and pro:EX1-2:GFP. In the elongation zone and maturation zone, pro:EX1-2:GFP showed slightly higher expression in vascular tissue (Figure S1A). However, there was no discernable difference in GFP intensity during lateral root organogenesis (Figure S1B). We also tested levels within the leaves using RT-qPCR and saw only modest changes between the two lines (Figure S1C). These data suggest that the first intron has a specific function in driving expression within the RAM.

### The first intron contains potential *cis*-elements

Our previous analysis revealed the first intron is vital for ARF7 transcriptional regulation in the root meristem. There are several well-studied examples within the plant kingdom in which transcription factors bind to regulatory elements located within introns to control expression. For instance, the second intron of *AGAMOUS* (*AG*) contains enhancer elements that are sufficient to cf. *AG* expression^36^ and binding sites within the second intron of *GLABRA3* (*GL3*) regulate its expression in trichomes.^33^ To explore whether the ARF7 first intron contained potential regulatory elements, we first examined conservation of the intronic sequence within the 1001 Genomes Sequencing project. The alignment of the sequences of first intron in these lines indicated a high degree of sequence conservation within the first intron, supporting the possibility that this region could have important binding sites.

**Figure 2:**
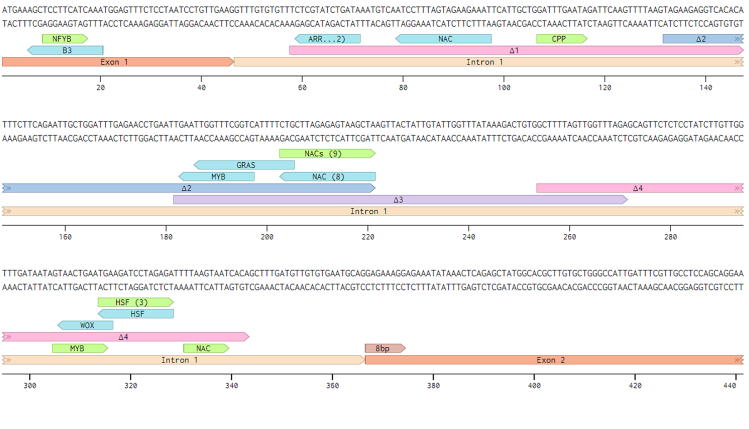
The first intron of ARF7 contains potential NAC and MYB transcription factor binding sites Genomic ARF7 sequence surrounding the 5′ end of the first exon, including the entire first intron and first 75bp of second exon. The 8bp of second exon was used in contstructs. The Intron is shaded in the lighter shade. Putative transcription factor binding motifs are annotated in this sequence (The green annotations are motifs binding to forward strand and the blue annotations are motifs binding to reverse). The number in brackets shows the number of members of each transcription factor family predicted to bind to these sites. Δ1 to Δ4 shows the 4 sections of intronic DNA deleted in the various constructs (see main text). Image generated using Benchling.

We next used the PlantRegMap (Plant Transcription Regulatory Map) which provides a high-quality resource of TFs and target genes, including a set of high-quality, non-redundant TF binding motifs derived from experiments and regulatory elements identified from high-throughput sequencing data.^37^ When a threshold *p* value<1e-4 was applied, this intron has 33 putative binding sites of 28 transcription factors, including 19 NAC family genes (Figure 2; Table S1). Simultaneously, we examined DNA affinity purification sequencing (DAP-seq) data, showing in-vitro-expressed TFs interacting with genomic DNA.^38^ These data identified four transcription factor gene families binding to the region of intron, these are bHLH, C2H2, NAC, and WRKY (Figure S2). All these NAC binding sites aligned with putative binding sites previously identified using the PlantRegMap analysis. To understand whether these selected binding sites could be unique and essential we studied their conservation across different *Arabidopsis thaliana* genetic variants. In this context, we first identified sequences from the first intron of *ARF7* from Arabidopsis 1001 genomes. Next, we MAFFT aligned all sequences, overlayed selected binding sites and visualized using Benchling to reveal that these TF binding sites were highly conserved.

### No single transcription factor binding site within the first intron is required to drive transcription in the primary root meristem

**Figure 3:**
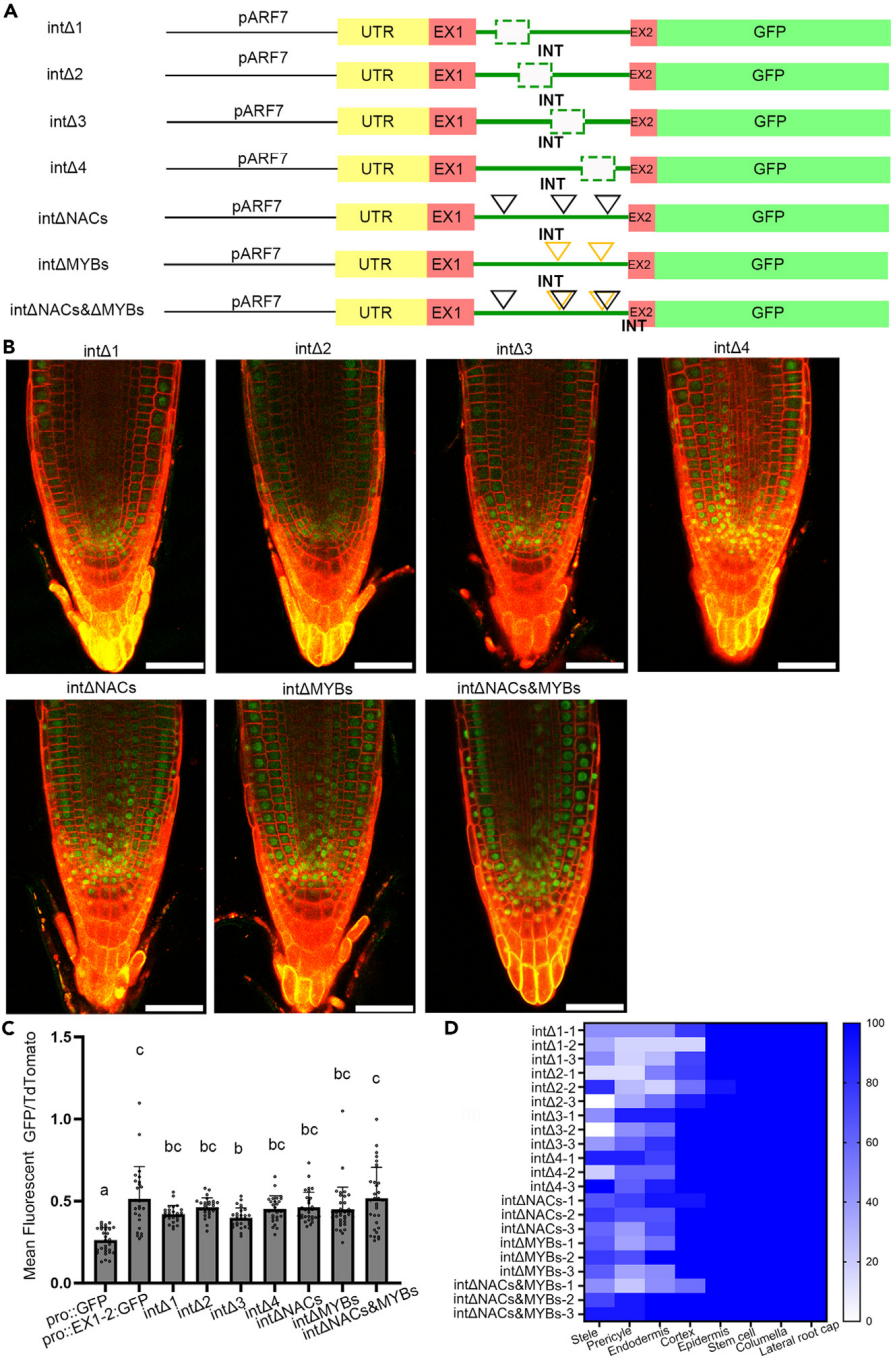
Functional Dissection of first intron reveals that no single regions is required to drive expression in the root apical meristem (A) Schematic illustration of intron dissection constructs. The deleted sequences are shown by a green dashed box. Black triangle boxes show the modified NAC binding sites and yellow triangle boxes show the modified MYB binding sites. (B) Confocal images showed *GFP* expression within the meristem of all the constructs. Scale bar is 50μm. (C) GFP level quantified by mean fluorescent GFP/td-Tomato. All constructs measured 3 independent transgenic lines, for intΔ1 each line including 9, 6, and 11 samples, for intΔ2 each line including 8, 11, and 9 samples, for intΔ3 each line including 9, 9, and 10 individual plants, for intΔ4 each line including 8, 11, and 8 samples, for intΔNACs each line including 12, 12, and 9 samples, for intΔMYBs each line including 11, 13, and 11 samples, for intΔNACs&MYBs each line including 9, 11, and 12 samples. Error bars are standard deviation of samples. Significant test was done by ANOVA with Tukey HSD. *p* < 0.05 (D) Heatmap showed the ARF7 expression frequency in different cell types in the RAM.

In order to functionally dissect the ARF7 intron and identify the location of key binding sequences, the intron was divided into four overlapping parts of approximately 90 bp, with each part containing several of the previously identified putative binding sites (Figure 2; Table S2). Each section was sequentially deleted in pro:EX1-2:GFP to give four constructs, named intΔ1-4, respectively (Figure 3A).

Plants transformed with these deletion constructs showed GFP signal within the RAM with strong expression around the stem cell niche (Figures 3B and 3D). Quantification of the GFP signal showed that all four deletion constructs were statistically distinct from the pro:GFP line (Figure 3B–3D).

These results showed no single binding site was sufficient to fully explain the role that the first intron has in mediating expression in the root tip, as no single deletion could remove GFP expression at the root tip. However, in our bioinformatic analysis we identified some classes of transcription factors with multiple binding sites. We focused on the transcription factors predicted to bind to multiple locations within the intron, namely the NAC and MYB (Myeloblastosis) transcription factors (Figure 2). The three NAC binding sites were in Δ1, the overlap of Δ2 and Δ3, and Δ4. The two MYB sites were located in Δ2 and Δ4 deletions.

We next investigated the importance of the three NAC binding sites by making three constructs in which one of NAC sites was mutated, termed intΔNAC1-3 (Figure S3A). However, no differences were observed in the GFP levels in these constructs (Figure S3B). We then made two constructs in which either both the MYB sites (intΔMYBs) or all three NAC sites (intΔNACs) were mutated and used this to drive GFP (Figure 3A). Again, we did not see significant changes in the GFP signal (Figures 3B and 3C). Finally, to test whether the NACs and MYB work together to promote transcription of *ARF7*, we made a new construct in which both MYB sites and all three NAC sites were mutated and used this to drive GFP. We still did not see significant changes in GFP signal (intΔNACs&MYBs) (Figures 3B and 3C).

### Intron-mediated enhancement may regulate *ARF7* expression at the root tip

Despite our extensive search for *cis*-regulatory elements within the first intron that may mediate expression of ARF7 in roots, we were unable to identify any binding sites that were required to drive expression at the RAM. This does not conclusively prove that no such elements exist but led us to consider alternative mechanisms. There are several examples in which introns play an important role in regulating transcription.^32^^,^^33^ In some cases, introns can increase the level of transcription, and in these examples greater quantities of mRNA are seen in constructs incorporating introns.^39^ This effect has been described as “intron-mediated enhancement” or IME.^40^ The role that mRNA-increasing introns play is not well understood, and the sequences associated with such introns are not well defined. However, there are a general set of rules for expression stimulating introns; these should be no more than 1kb from the transcriptional start site and must be in a transcribed region. For introns providing an IME, deletion experiments do not provide a good way to identify regulatory elements as in cases where this has been tried (e.g., UBQ10) it is likely that active sequences are distributed through the intron.^41^ The IMEter computational tool^42^ allows the degree of similarity of a given intron to other promoter-proximal introns to be calculated. The rationale is that only promoter-proximal introns are likely to play a role in IME. When the first intron of ARF7 is run through this tool it comes out with a strong enrichment for promoter-proximal elements, ranking within the top 94th percentile of Arabidopsis introns. IMEter scores in the 90^th^ percentile and higher are generally considered to be strong candidates for IME. For example, a study of the most active genes in soybean revealed that all the genes identified as producing the highest amount of mRNA contained an intron with an IMEter score in the 92^nd^ percentile or higher.^40^^,^^43^ Given the prediction with IMEter, we next considered whether IME could play a role in controlling ARF7 expression in the primary root meristem.

**Figure 4:**
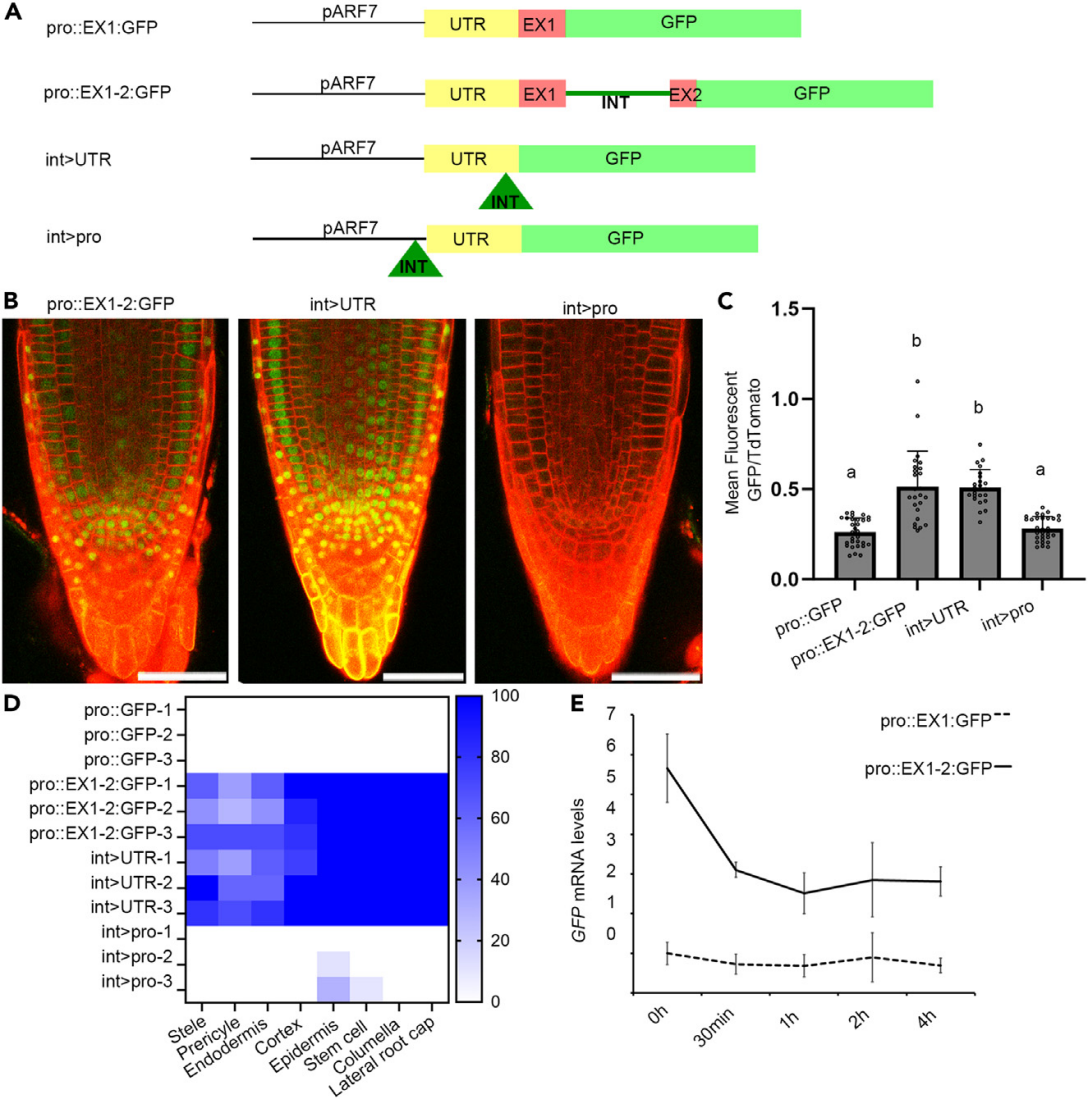
The first intron only affected expression when it was present in the transcribed mRNA (A) Schematic illustration of different intron location, the green triangle showed first intron insertion. (B) Confocal images showed *ARF7* expression within the meristem of all the constructs. Scale bar is 50μm. (C) ARF7 expression level quantified by mean fluorescent GFP/td-Tomato. All constructs measured 3 independent transgenic lines, for int>UTR each line including 8, 5 and 10 samples, for int>pro each line including 11, 9 and 10 samples. Error bars are standard deviation of samples. Significant test was done by ANOVA with Tukey HSD. *p* < 0.05 (D) Heatmap showed the ARF7 expression frequency in different cell type in RAM. (E) ARF7 expression level quantified by RT-qPCR in 2mm root tips after Actinomycin D treatment. Error bars are standard deviation of three independent transgenic lines.

A discriminating characteristic of IME is that it requires the intron to be transcribed, whereas enhancers do not need to be present in the mRNA.^44^ To test this, we constructed two new reporters in which we moved the intronic sequences either to the 5′ UTR (int>UTR) or upstream of the UTR (int>pro). Our rationale was that if the intron mediated its effect on *ARF7* transcription via an enhancer sequence then both constructs would be sufficient to drive expression in the RAM. However, if the intron mediated its effect on expression through IME, then only the construct in which the intronic sequences are located in the transcribed region should drive expression at the root meristem. We designed the first construct such that the intron was inserted −46 nt upstream from the ATG translational start site (Figure 4A). To ensure correct splicing of this intron, we also inserted exon-intron junctions on both sides. To verify that this new intron was correctly spliced, we used RT-PCR with a pair of primers located within the 5′UTR and the GFP coding sequence (Figure S4A). We compared the size of amplified band between several lines. In plants with the pro:EX1-2:GFP construct, the size of the fragment with splicing or without splicing should be 332bp or 652bp. For the int>UTR lines, the fragment spliced or without splicing should be 312bp or 632bp. A fragment of around 300bp was present in transgenic lines harboring either the pro:GFP or pro:EX1:GFP constructs, thus showing correct transcript splicing of both constructs (Figure S4B). These results were confirmed by Sanger sequencing of amplified fragments. Plants harboring the int>UTR showed expression patterns in the RAM that were indistinguishable from (pro:EX1-2:GFP) (Figure 4B). In the second construct (int>pro) the intronic sequences were located −622 nt upstream from the ATG translational start site outside of the translated region (and 13 bp from the transcriptional start site). Plants harboring the int>pro showed similar levels of GFP fluorescence in the root tip (Figure 4B) but retained expression in lateral roots (Figure S1B).

**Figure 5:**
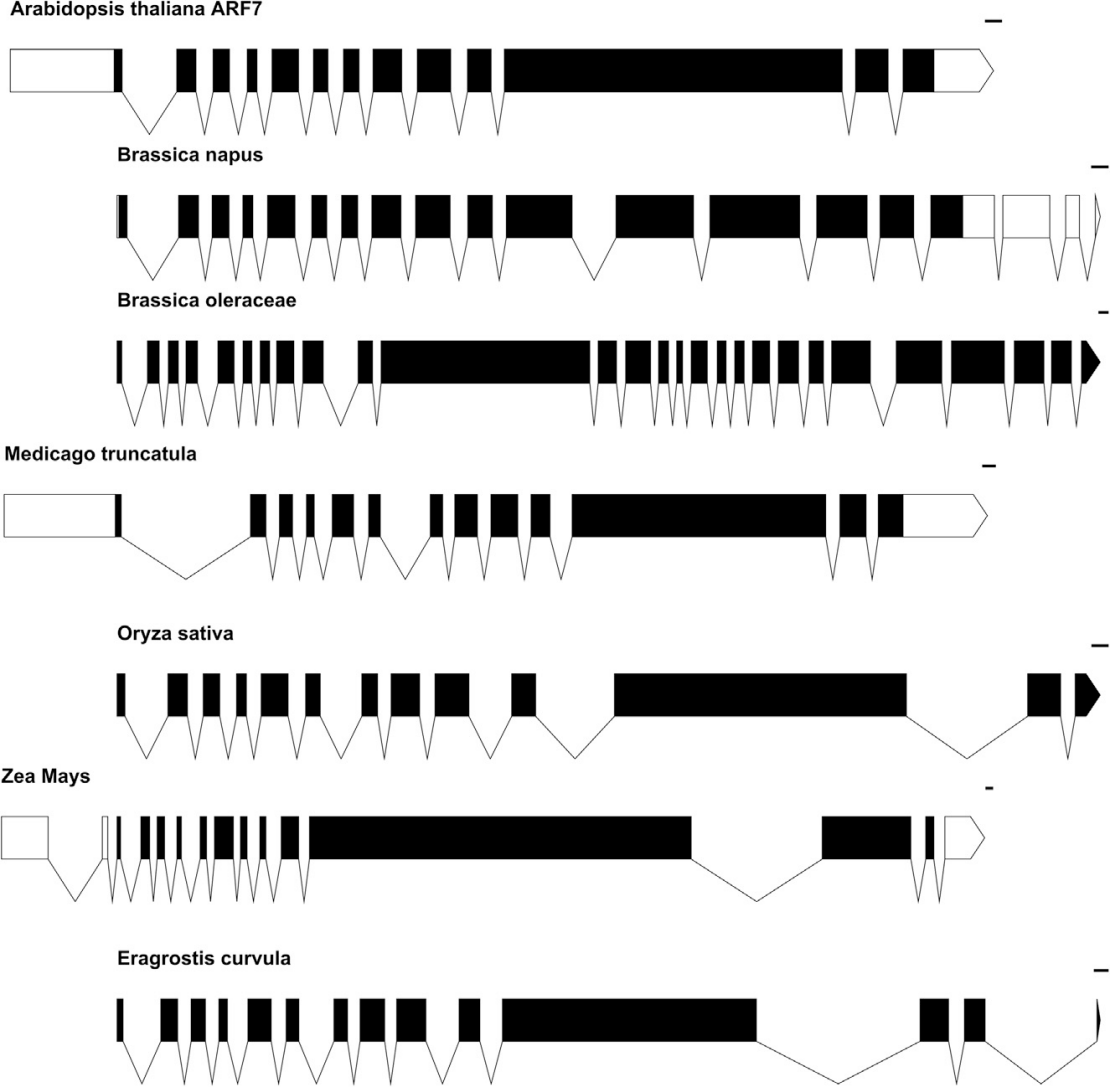
Putative ARF7 orthologues in six diverse species all contain a sizable first intron 40-46bp from the translational start site, related to main text Exon-intron structures of putative *ARF7* orthologues in diverse flowering plants. Arabidopsis ARF7 was subjected to a BLAST search and a sample of species that contained one-to-one orthologues were selected. In this selection of three monocots and three dicots, the first intron in the coding sequence was larger than average and located at an identical place. *Zea mays* has two introns within the 5′ UTR so that the conserved third intron shows corresponds with the first in other species. Schematic diagrams have been aligned based on the translational start site. The scale bars show 100 bp.

Collectively the fact that the intronic sequences needed to be present in a region of the promoter that was transcribed, supports a model in which the intron exerts its effect on ARF7 expression via IME rather than through the existence of specific enhancer elements, as the enhancers were present in both constructs. The importance of the first intron in Arabidopsis *ARF7* led us to question whether this feature was conserved in other plant species. We took a sample of three monocot and three other dicot species where we found one to one ARF7 orthologues and investigated the intron-exon structure in these lines. All these species contained a sizable intron with a highly conserved junction 40–46 bp downstream of the translational start (Figures 5, S5, and S6, Tables S3 and S4).

### The first intron does not affect mRNA stability

The mechanism by which IME operates is yet to be fully determined. In the case of *ARF7*, our results show greater mRNA levels in lines containing the first intron. We therefore reasoned that the first intron may affect either transcriptional initiation or mRNA stability. To test the latter, we treated the pro:EX1:GFP and pro:EX1-2:GFP lines with Actinomycin D. This prevents the syntheses of new mRNA.^45^ We then quantified the levels of *GFP* mRNA via RT-qPCR over a period of 4 h. As expected, the intron containing line (pro:EX1-2:GFP) had a higher initial mRNA level than the line without intron (pro:EX1:GFP) (Figure 4E). For the line without intron, the mRNA degraded slowly, however, for the line with intron, there was a rapid reduction in mRNA level within 30 min but this then became more stable over longer periods. These dynamics do not support a hypothesis in which the first intron enhances mRNA stability but rather suggests the intron increases the production rate of ARF7 mRNA in the root.

## Discussion

In this study we developed a series of constructs which collectively indicated that the first intron of ARF7 is required for broad expression in RAM. It was previously shown that sequences in the 5′ end of the *ARF7* mRNA located between the first exon and the start of the second exon play an important role in transcription regulation in the root.^20^ We show that the sequences exerting this effect lie within the first intron and are unlikely linked to either the first exon or the start of the second exon. We generated multiple constructs affecting either putative or demonstrated transcription factors binding sites within this intron or harboring partial deletions. In both cases the mutated sequences were capable of driving expression at the root tip. While it is possible that we have missed non-predicted regulatory sequences acting as enhancers within the intron, the extent of our constructs supports a hypothesis is which there is no single enhancer element within the intron that solely drives expression at the root tip.

This led us to consider an alternative regulatory mechanism by which the first intron could influence the transcription of ARF7 via intron-mediated enhancement. As the position of the intron plays a crucial role in determining transcript levels in cases of intron-mediated enhancement,^40^ we explored whether the first intron exerted the same effect when it was present either within or upstream of the transcribed sequence. We observed a broad expression of ARF7 in the RAM when the intron is present in the UTR but not if it is upstream of the transcriptional start. We also showed that when incorporated within the UTR the intron was correctly spliced out, thus ruling out a stabilization of the mRNA through addition of the first intron sequence to the final mRNA. Collectively our results are consistent with a mechanism in which the intron needs to be present in the mRNA to boost expression in the root. This is consistent with the idea that the first intron may exert its role on *ARF7* expression via IME rather than by the presence of specific enhancer elements. It also raises the intriguing question of whether the *ARF7* intron would exert a similar role if it was introduced to a different gene.

The effect of IME on boosting gene expression has been documented on many occasions.^32^^,^^33^^,^^46^ There are examples of introns causing increased transcription,^47^ enhancing mRNA stability,^48^ and boosting translation.^49^ To test whether ARF7 first intron had a role in regulating mRNA stability we measured the transcript levels of GFP in two constructs (one with and one without the first intron). After an initial decrease in the level of mRNA of the intron containing line, we did not see a clear difference in mRNA degradation between the two constructs. This suggests that in this context IME does not play a role in regulating mRNA stability. Our results are consistent with a role for the ARF7 first intron in boosting transcription levels, perhaps through transcriptional re-initiation. While there are numerous examples of IME within the plant kingdom these generally involve genes expressed at high levels with broad expression patterns, including genes such as UBQ10.^41^ Our study of ARF7 provides a rare example of what could be tissue-specific IME. The observation that *ARF7* orthologues in diverse flowering plants also share a similar intron-exon structure at the 5′- end of the gene hints at the possibility of a conserved role of introns in regulating *ARF* expression in a wider range of species. Previously, the effect that IME has on tissue-specific promoters had been investigated by including the first UBQ10 intron in a suite of tissue specific and non-specific promoters. Incorporation of this intron causing tissues-specific promoters to be expressed more widely.^50^ If ARF7 expression is controlled by IME, this raises intriguing questions regarding how it shapes expression in some cell types and not others. Specifically, why the intron exerts this effect in primary root tips, but not in other tissues such as lateral root primordia and shoots.

### Limitations of the study

The role of the first intron has been investigated in the context of its regulation of GFP expression. A limitation of this study is that these promoter/intron variants have not been used to drive ARF7 cDNA and look for complementation of ARF7 mutants. This was our intention, but while cloning and transformation of the GFP reporters was highly efficient, we repeatedly failed to transform constructs including the ARF7 cDNA into plants. Transforming ARF7 is clearly possible, as 35S::ARF7 lines have been published.^26^ However, we tried the transformation 3 times, and failed to recover independent transgenics. This leads us to suspect that the presence of ARF7 cDNA is either inhibitory to the insertion of the transgene into Arabidopsis or often lethal to plant growth.

## STAR★Methods

### Key resources table

**Table:** 

REAGENT or RESOURCE	SOURCE	IDENTIFIER
**Bacterial and virus strains**		

*E. coli* DH5α	In house stock	N/A
Agrobacterium GV3101	In house stock	N/A

**Chemicals, peptides, and recombinant proteins**		

Murashige & Skoog Medium, basal salt mixture	Sigma-Aldrich	M5524
Agar	Sigma-Aldrich	A1296
Hygromycin B (50 mg/mL)	Thermo Fisher	Cat#10687010
Actinomycin D	Sigma-Aldrich	A1410
Silwet L-77	De Sangosse	N/A

**Critical commercial assays**		

Gibson Assembly® Master Mix	NEW ENGLAND BioLabs	E2611L
Golden Gate Assembly Kit (BsaI-HF® v2)	NEW ENGLAND BioLabs	E1601L
Q5® Site-Directed Mutagenesis Kit	NEW ENGLAND BioLabs	E0552S
GreenGate Cloning System	Addgene	Kit #1000000036
RNeasy Plant Mini Kit	QIAGEN	Cat# 74904
RevertAid First Strand cDNA Synthesis Kit	Thermo Fisher	K1621
SensiMix™ SYBR® Hi-ROX Kit	Bioline	QT605-05

**Deposited data**		

Microscopy data	This paper; Mendeley Data	https://doi.org/10.17632/4t9twsz9bg.2

**Experimental models: Organisms/strains**		

*Arabidopsis Thaliana* Columbia-0	In house stock	N/A
*Arabidopsis Thaliana*: pro:GFP	This study	NASC ID: N2112003
*Arabidopsis Thaliana*: pro:EX1:GFP	This study	N/A
*Arabidopsis Thaliana*: pro:EX1-2:GFP	This study	NASC ID: N2112004
*Arabidopsis Thaliana*: intΔ1	This study	N/A
*Arabidopsis Thaliana*: intΔ2	This study	N/A
*Arabidopsis Thaliana*: intΔ3	This study	N/A
*Arabidopsis Thaliana*: intΔ4	This study	N/A
*Arabidopsis Thaliana*: intΔNACs	This study	N/A
*Arabidopsis Thaliana*: intΔMYBs	This study	N/A
*Arabidopsis Thaliana*: intΔNACs&ΔMYBs	This study	N/A
*Arabidopsis Thaliana*: intΔNAC1	This study	N/A
*Arabidopsis Thaliana*: intΔNAC2	This study	N/A
*Arabidopsis Thaliana*: intΔNAC3	This study	N/A
*Arabidopsis Thaliana*: int>pro	This study	N/A
*Arabidopsis Thaliana*: int>UTR	This study	NASC ID: N2112005

**Oligonucleotides**		

Primers (Table S5)	This study	N/A

**Recombinant DNA**		

pGGA-ARF7proS	This study	Addgene 218563
pGGA-ARF7UTRINT	This study	Addgene 218564
pGGA-ARF7INTUTR	This study	Addgene 218565
pGGB-EX1	This study	Addgene 218566
pGGB-EX1-2	This study	Addgene 218567
pGGB-EX1-2 (intΔ1)	This study	Addgene 218568
pGGB-EX1-2 (intΔ2)	This study	Addgene 218569
pGGB-EX1-2 (intΔ3)	This study	Addgene 218570
pGGB-EX1-2 (intΔ4)	This study	Addgene 218571
pGGB-EX1-2 (intΔNACs)	This study	Addgene 218572
pGGB-EX1-2 (intΔMYBs)	This study	Addgene 218573
pGGB-EX1-2 (intΔNACs&ΔMYBs)	This study	Addgene 218574
pGGB-EX1-2 (intΔNAC1)	This study	Addgene 218575
pGGB-EX1-2 (intΔNAC2)	This study	Addgene 218576
pGGB-EX1-2 (intΔNAC3)	This study	Addgene 218577
pro:GFP	This study	Addgene 218563
pro:EX1:GFP	This study	Addgene 218564
pro:EX1-2:GFP	This study	Addgene 218565
pro:EX1-2(intΔ1):GFP	This study	Addgene 218566
pro:EX1-2(intΔ2):GFP	This study	Addgene 218567
pro:EX1-2(intΔ3):GFP	This study	Addgene 218568
pro:EX1-2(intΔ4):GFP	This study	Addgene 218569
pro:EX1-2(intΔNACs):GFP	This study	Addgene 218570
pro:EX1-2(intΔMYBs):GFP	This study	Addgene 218571
pro:EX1-2(intΔNACs&ΔMYBs):GFP	This study	Addgene 218572
pro:EX1-2(intΔNAC1):GFP	This study	Addgene 218573
pro:EX1-2(intΔNAC2):GFP	This study	Addgene 218574
pro:EX1-2(intΔNAC3):GFP	This study	Addgene 218575
int>pro:GFP	This study	Addgene 218576
int>UTR::GFP	This study	Addgene 218577

**Software and algorithms**		

ImageJ	Schneider et al.^51^	https://imagej.nih.gov/ij/
Leica LAS X	Leica	https://www.leica-microsystems.com/products/microscope-software/p/leica-las-x-ls
Benchling	Benchling	https://www.benchling.com
GraphPad Prism 10	Graphpad	https://www.graphpad.com/features
IBM SPSS Statistics 29	IBM	https://www.ibm.com/products/spss-statistics
MEGA-X	megasoftware	https://megasoftware.net/
Jalview	Jalview	https://www.jalview.org/

### Resource availability

#### Lead contact

Further information and requests for resources and reagents should be directed to and will be fulfilled by the lead contact, Anthony Bishopp (Anthony.bishopp@nottingham.ac.uk).

#### Materials availability

• Destination plasmids generated in this study have been deposited to Addgene. All intermediate modules are available on request from the lead contact.
• The most useful Arabidopsis lines generated in this study have been deposited to NASC. All other lines are available on request from the lead contact.

#### Data and code availability

• The microscopy data used for quantification is deposited on Medeley Data https://data.mendeley.com/datasets/4t9twsz9bg/1. All other datasets reported in this work are available from the lead contact on request.
• This paper does not report original code.
• Any additional information required to reanalyze the data reported in this paper is available from the lead contact upon request.

### Experimental model and study participant details

#### Plant material and growth conditions

All lines generated in this study were in the Arabidopsis thaliana Col-0. The seedlings were grown at 21^o^C. The growth conditions for individual experiments are listed in the methods.

### Method details

#### Constructs of transcriptional reporter lines

All constructs were produced using the DEAL vector system.^35^ This is a modified version of the GreenGate cloning system^34^ to include a red plasma membrane marker to highlight anatomy and serve as an internal control to ensure that all transgenes were expressed. All modules and primers are shown in Tables S5 and S6.

For reporter lines, the promoter and intron were cloned respectively into A and B entry modules. In line int>UTR and int>pro lines, the promoter A modules were edited to insert the intronic sequences within the UTR or upstream of the UTR using the Gibson cloning system.^52^ The Site-Directed Mutagenesis Kit (NEW ENGLAND BioLabs) was used to introduce polymorphisms affecting specific binding sites within intron constructs by editing the EX1-2 B module. The DNA sequences knocking out multiple MYB and NAC binding sites were synthesized by Eurofins. NAC and MYB mutations were mutated by exchanging 2bp within the core binding motif, which is shown in Table S2. 2bp in the core TF binding sequence were altered based on the TFs position weight matrices and TFs flexible models provided by JASPAR.^53^^,^^54^ Combining the position information from JASPAR and the sequences from PlantRegMap, we selected motifs to target to modulate the interaction of NAC and MYB proteins with the intronic sequence. Entry modules recombined in order, with GFP:NLS (C module) and D-dummy, RBCS terminator and HygromycinR (for plant selection) into the DEAL vector.

All destination plasmids were assembled by Golden Gate Assembly Kit (NEW ENGLAND BioLabs). These constructs were transformed in *Agrobacterium tumefaciens* GV3101 via electroporation and transformed into Col-0 via the floral dip method,^55^ which is dipping Arabidopsis inflorescences for a 1min into a 5% sucrose solution containing 0.05% Silwet L-77 and resuspended Agrobacterium. Transgenic T0 seeds were plated on 1% agar containing 1/2 MS medium and 20 μg/L Hygromycin B. Only T2 lines with single locus insertions were analyzed, as judged by a 3:1 resistance to hygromycin. After a 2days stratification period, seeds were grown in 21°C with 6 h light, 48 h dark and 24 h light.^56^

#### Confocal analysis

For the primary root microscopy, 3 independent T2 lines were grown on 1/2 MS medium supplemented with 1% agar in 16h light/8h dark conditions for 5 days (for primary roots) or 8 days (for lateral roots). The roots were examined in the TCS-SP8 confocal microscope (Leica) with excitation at 488 nm and emission at 496–550 nm for GFP, excitation at 560nm and emission at 590–700 nm for tdTomato. Fluorescent signal was quantified in ImageJ for over 5 lines (including both hetero and homozygous individuals, the numbers of lines were indicated in figure legends) by selecting a rectangle (130 × 190 micrometers) with the longest side parallel to the root axis. Fluorescence was measured as mean fluorescence intensity of GFP and calculated as a ration of GFP to TdTomato.

#### Expression analysis by qRT-PCR

For reporter lines analysis, 3 independent T2 plants were grown on 1/2 MS medium supplemented with 1% agar in 16h light/8h dark conditions for 5 days (for root meristem) or 10 days (for leaf). The root meristem sample of each T2 line was obtained by dissecting and pooling 2mm root tips from 50 individual plants. The leaf sample of each line contained 3 plant leaves. Each line including 3 technical replicates. For mRNA stability analysis, 5 days roots were treated with 100μg/ml Actinomycin D in 1/2 MS medium, for 30min, 1h, 2h and 4h. RNA was collected from T3 homozygous lines. Each line has 3 biological repeats and each sample including 50 2mm root tips. Each line including 3 technical replicates. The Qiagen RNeasy Plant Mini Kit was used for RNA extraction. The cDNA was synthesized using RevertAid First Strand cDNA Synthesis kit (Thermo Scientific) with 200ng RNA and diluted 3:20 for qRT-PCR reaction. The SensiMix SYBR Hi-Rox Mastermix (BIOLINE) was used for qRT-PCR reaction. The control gene was *UBC21* (At5g25760). Primers were showed in Table S5.

#### Methodology for motif analysis

We first used AT5G20730.1 as a representative model to obtain the 1st intron sequence (Chromosome 5: 7021458.7021139; minus strand; reverse complemented) of ARF7. Next, we scanned this input sequence using PlantRegMap tool^37^^,^^57^^,^^58^ for the presence of any manually curated 674 non-redundant and high-quality binding motifs derived from various experiments, literature and ChIP-seq datasets. We specifically used the Binding Site Enrichment tool (with settings: species = Arabidopsis thaliana and Threshold *p*-value ≤ 1e-4) that uses modified FIMO (Find Individual Motif Occurrences) to search TF binding sites in the input sequence.

To cross-validate the above predictions, we used recent DNA affinity purification sequencing data (DAP-seq) to scan input region (Chromosome 5: 7021458.7021139) for peaks (with FRiP ≥ 5% i.e., fraction of reads in peaks) of 46 different transcription factors. The overlap between predicted and Dap-Seq derived TF binding sites (termed as selected binding sites) were considered for further analysis.

#### Sequences and alignment

To illustrate conservation of regions in the intron and first two exon sequences of ARF7 across different plant species, known orthologues were selected from the orthologue table of AT5G20730.3 in EnsemblPlants. These selections were made to include primarily agriculturally important species, and representation of both monocots and dicots. Multiple sequence alignment of these sequences were performed using MUSCLE in MEGA-X (version 10.2.1), with alignment figure constructed using Jalview software (version 2.11.3.2).

### Quantification and statistical analysis

T test statistical analyses were performed in Excel. ANOVA statistical analyses were performed in SPSS 29. The precise statistical tests can be found in the corresponding Figure legend.

## Supplemental information

Document S1. Figures S1‒S6 and Tables S1–S6
