# Supplementary material for: The first intron of ARF7 is required for expression in root tips
Source: iScience. 2024 May 8;27(6):109936. doi: 10.1016/j.isci.2024.109936 (PMC11145351; doi:10.1016/j.isci.2024.109936)
Supplement: Document S1. Figures S1‒S6 and Tables S1–S6 [file mmc1.pdf]

iScience, Volume *27*

Supplemental information

The first intron of ARF7

is required for expression in root tips

Jingyi Han (揿静宜), Thomas Welch, Ute Voß, Teva Vernoux, Rahul Bhosale, and Anthony Bishopp

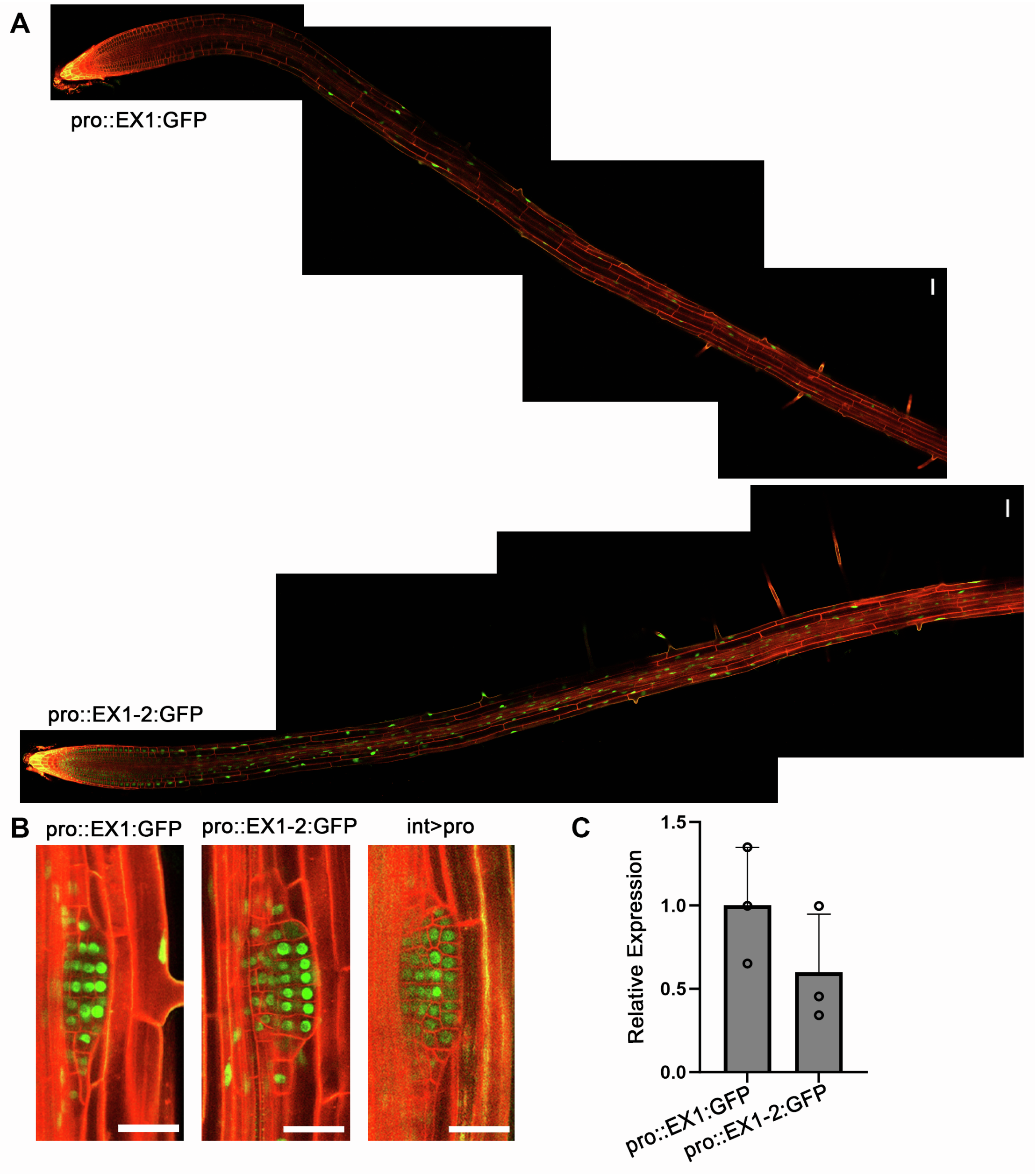

Figure S1: *ARF7* has similar expression pattern in other tissues regardless of the presence of the intron, related to Figure 1. A.) Confocal images showing *ARF7* expression with or without intron constructs in primary roots. ARF7 is expressed in mature tissues in the vascular and epidermis. The scale bar is 50μm. B.) Confocal images showed *ARF7* expression for constructs with or without intron in lateral root primordia. Scale bar is 50μm. C.) qRT-PCR result showed GFP expression level with or without intron constructs in whole leaves. $p<0.05$. Significant test was done by T-test and shown not to be significant. Error bars are standard deviation of three independent transgenic lines.

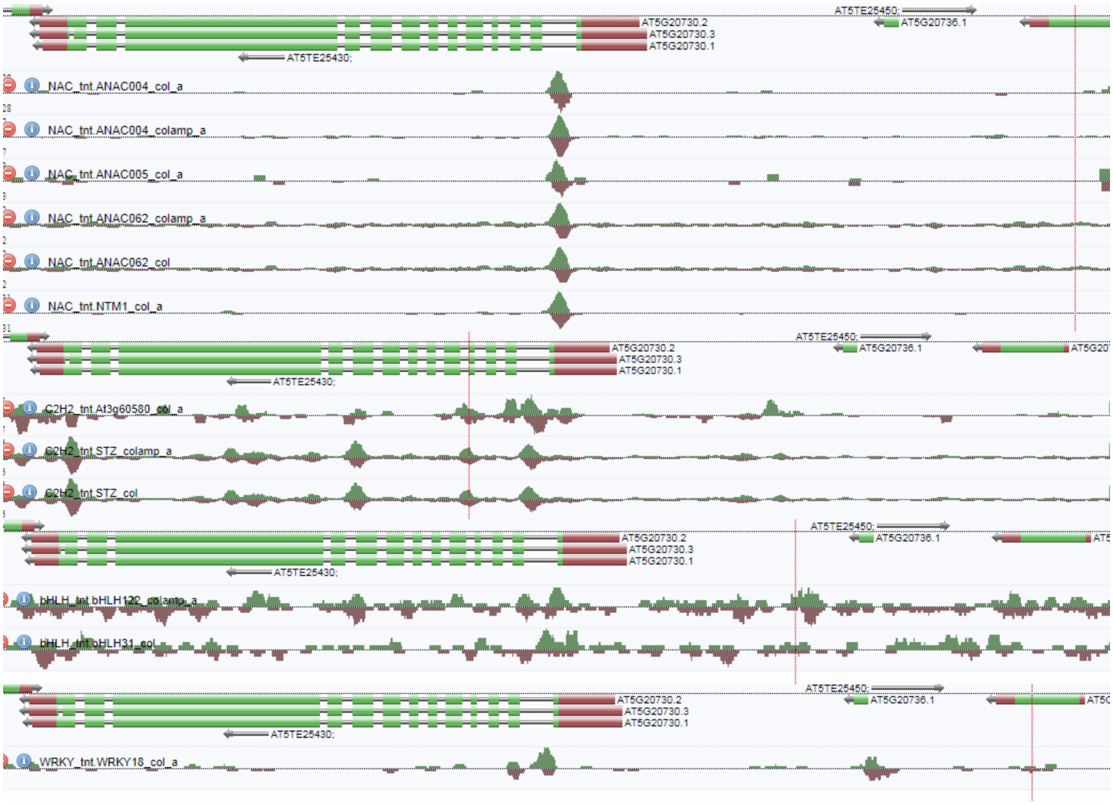

Figure S2: DAP-seq showed potential TFs occupied to first intron, related to Figure 2. The red and green bars represent the *ARF7* locus. The green and red peaks showed the DAP-seq peaks of TFs.

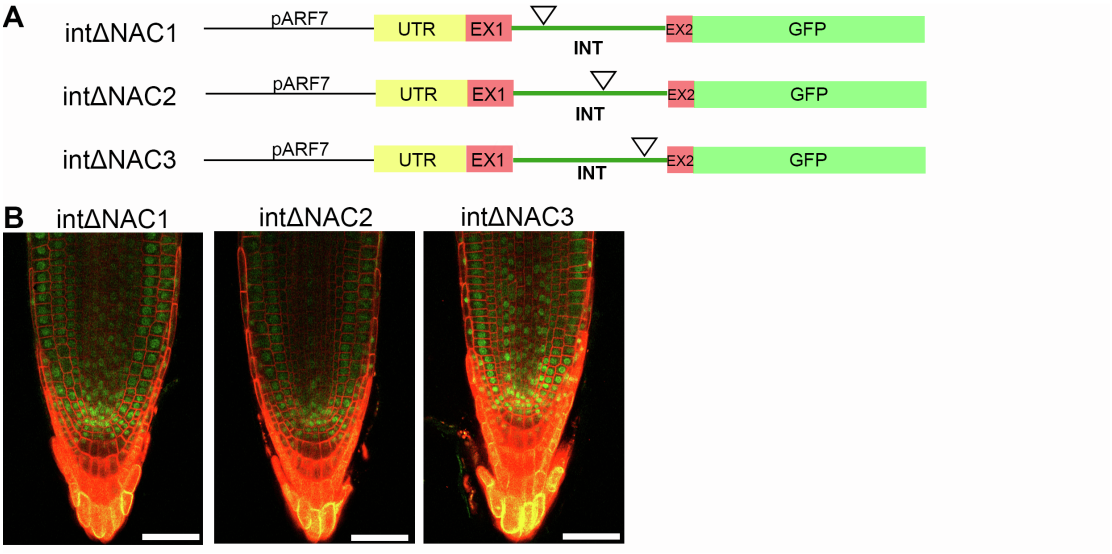

Figure S3: Functional Dissection of first intron reveals that no single NAC site is required to drive expression in the root apical meristem, related to Figure 3.
A.) Schematic illustration of intron dissection constructs. The deleted sequences are shown by a green dashed box. Black triangle boxes show the modified NAC binding sites. B.) Confocal images showed *ARF7* expression within the meristem of all the constructs. Scale bar is 50μm.

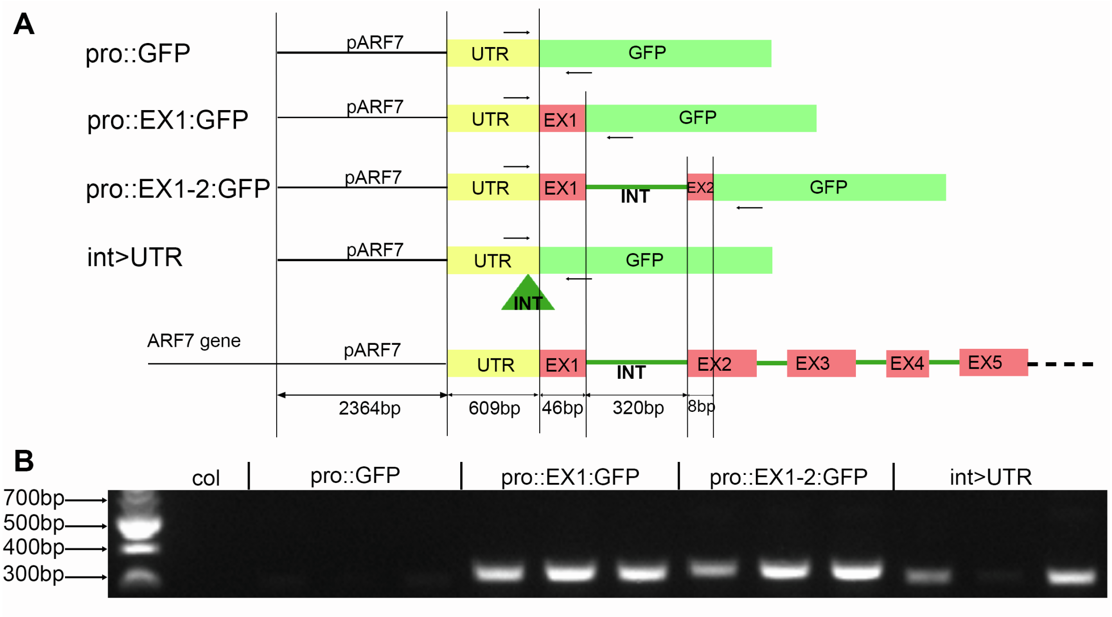

Figure S4: The first intron of *ARF7* was correctly spliced in all constructs, related to Figure 4. A.) The location of primers was indicated as arrow. B.) RT-PCR showed splicing of ARF7 with different length promoters. Each construct had three independent lines. Expected band sizes were as follows: pro::GFP is 300bp; pro::EX1:GFP is 323bp; pro::EX1-2:GFP is 332bp; int>UTR is 312bp.

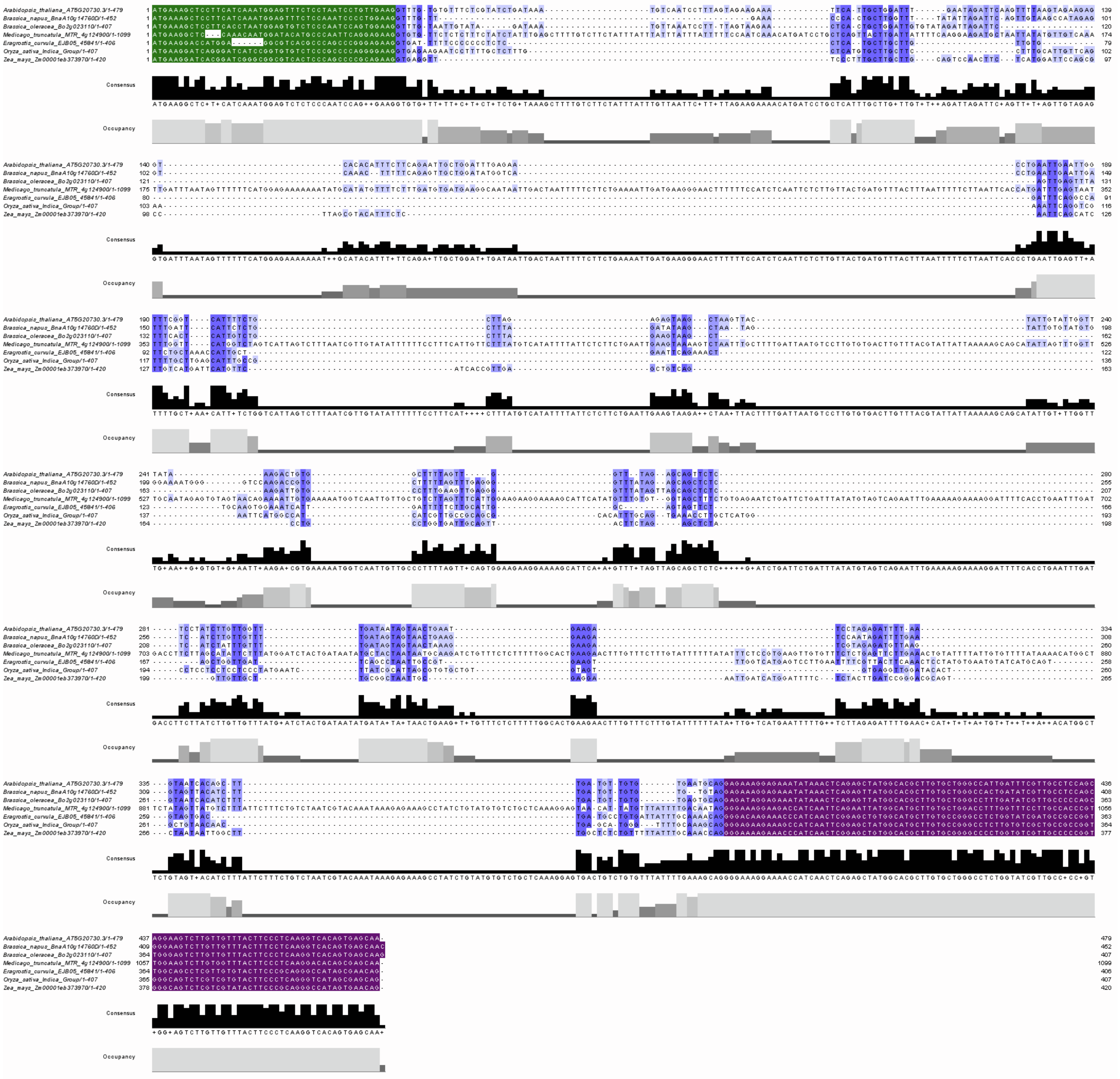

Figure S6: Alignment of the first Arabidopsis *ARF7* intron with orthologues in six diverse species, related to Figure 5. Sequences from the first exon within the coding sequence are shown in green with the following exon in purple.

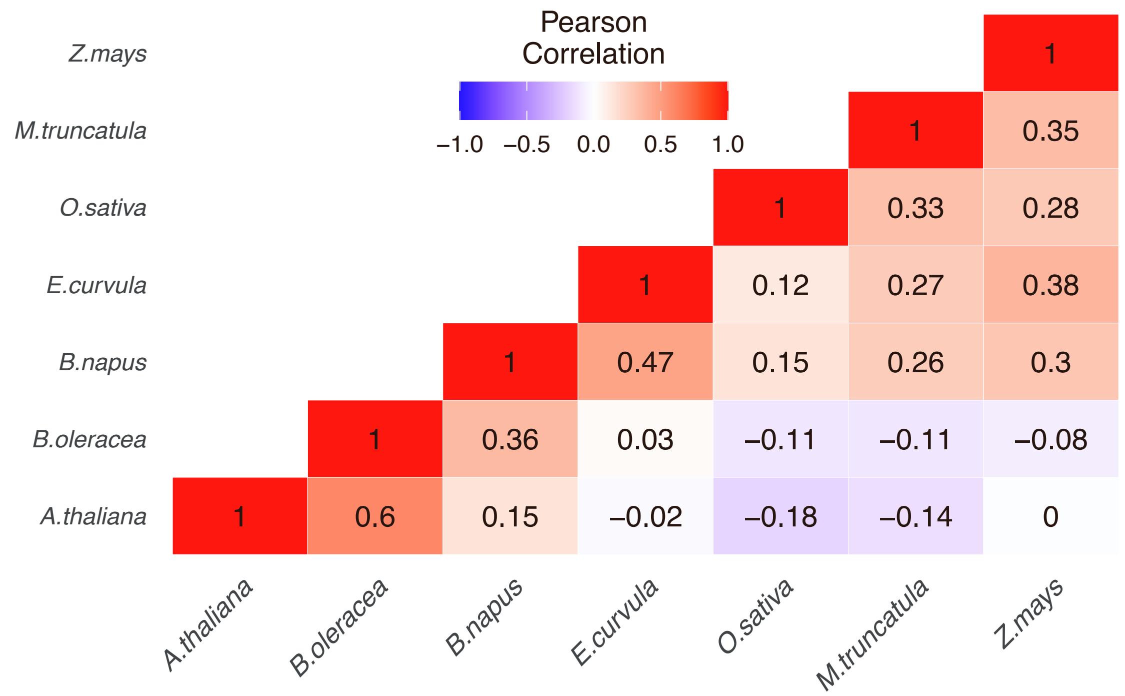

Figure S6. Correlation matrix of counts for putative binding sites within the first intron of ARF7 orthologues from diverse plant species, related to Figure S5. Matrix shows degree of correlation according to number of different TF families predicted within intronic sequence for each species (raw data shown in Table S4). Pairs with coefficients closer to one may therefore be considered more similar than pairs with lower values.

Supplemental Table 1: Potential transcription factor binding sites within the first intron, related to Figure 2.

The 33 putative binding sites of 28 Transcription Factors in intron. A threshold p-value<1e-4 was applied in PlantRegMap.

Gene	Family	Name	score	p-value	matched sequence
AT2G01760	ARR-B	RR14	10.8605	6.74E-05	AGATACGA
AT5G58080	ARR-B	RR18	10.5474	9.28E-05	TATCAGATACGAG
AT5G18090	B3	AT5G18090	11.8904	7.00E-05	TTTGATGAAGGAGCT
AT4G29000	CPP	AT4G29000	11.6224	7.35E-05	GATTTGAATA
AT5G05090	G2-like	AT5G05090	11.4894	5.84E-05	TCAGATACGAG
AT2G01570	GRAS	RGA1	11.9571	3.34E-05	CAGAAAATGACCGAAACCAA
AT1G46264	HSF	HSFB4	7.43836	5.58E-05	TGAAGATCCTAGAGA
AT2G41690	HSF	HSFB3	10.3797	7.86E-05	TCTCTAGGATCTTCA
AT3G22830	HSF	HSFA6B	9.54688	8.10E-05	TGAAGATCCTAGAGA
AT5G16820	HSF	HSF3	11.0156	4.79E-05	GAAGATCCTAGA
AT2G32460	MYB	MYB101	11.9062	3.83E-05	GACCGAAACCAATTC
AT5G06100	MYB	MYB33	11.4844	6.70E-05	GTAACTGAATG
AT4G01550	NAC	NAC069	12.1094	4.10E-05	AGCTTACTCTCTAAG
AT2G27300	NAC	NTL8	12.4844	2.35E-05	ATTTCTTCTACTAAAGGAT
AT1G02250	NAC	NAC005	9.29688	9.19E-05	CTGCTTAGAGAGTAAGCTA
AT1G02230	NAC	NAC004	13.9062	1.24E-05	CTTACTCTCTAAGC
AT1G12260	NAC	NAC007	11.2344	6.05E-05	CTTACTCTCTAAGCA
AT3G49530	NAC	NAC062	14.1562	1.29E-05	CTTACTCTCTAAGCA
AT4G36160	NAC	NAC076	11.0469	6.80E-05	CTTACTCTCTAAGCA
AT1G12260	NAC	NAC007	11.4375	5.43E-05	CTTAGAGAGTAAGCT
AT3G49530	NAC	NAC062	13.640	1.79E-	CTTAGAGAGTAAGCT

			6	05	
AT4G36160	NAC	NAC076	11.1875	6.34E-05	CTTAGAGAGTAAGCT
AT1G32870	NAC	NAC13	8.35938	6.80E-05	CTTAGAGAGTAAGCTA
AT3G10480	NAC	NAC050	5.54688	9.12E-05	CTTAGAGAGTAAGCTA
AT5G46590	NAC	NAC096	10.0312	6.86E-05	CTTAGAGAGTAAGCTA
AT5G66300	NAC	NAC105	12.5469	2.31E-05	CTTAGAGAGTAAGCTA
AT4G01540	NAC	NTM1	13.6562	1.70E-05	GCTTACTCTCTAAGC
AT4G01540	NAC	NTM1	9.625	6.54E-05	GCTTAGAGAGTAAGC
AT2G46770	NAC	NST1	12.3438	2.98E-05	TAGCTTACTCTCTAAGC
AT1G02250	NAC	NAC005	11.2344	4.58E-05	TAGCTTACTCTCTAAGCAG
AT4G35580	NAC	NTL9	15.0577	2.49E-05	TTAAGTAAT
AT2G38880	NF-YB	NF-YB1	3.72603	8.33E-05	TCCTTCATC
AT2G17950	WOX	WUS	10.9747	7.05E-05	TCATTCAGTTA

Supplemental Table 2. Mutated sequences used to remove NAC and MYB sites, related to Figure 3. The grey italicised letters are deleted base pairs. The red letters are mutated base pairs.

Name	Sequence
Orginal Intron	GTTTGTGTGTTTCTCGTATCTGATAAATGTCAATCCTTTAGTAGAAGAAATTCATTGCTGGA<br>TTTGAATAGATTCAAGTTTTAAGTAGAAGAGGTCACACATTTCTTCAGAATTGCTGGATTT<br>GAGAACCTGAATTGAATTGGTTTCGGTCATTTTCTGCTTAGAGAGTAAGCTAAGTTACTAT<br>TGTATTGGTTTATAAAGACTGTGGCTTTTAGTTGGTTTAGAGCAGTTCTCTCCTATCTTGTT<br>GGTTTGATAATAGTAACTGAATGAAGATCCTAGAGATTTTAAGTAATCACAGCTTTGATGTT<br>GTGTGAATGCAG
Δ1	GTTTGTGTGTT(*TCTCGTATCTGATAAATGTCAATCCTTTAGTAGAAGAAATTCATTGCTGG*<br>*ATTTGAATAGATTCAAGTTTTAAGTAGAAGAGGTCACACA*)TTTCTTCAGAATTGCTGGATT<br>TGAGAACCTGAATTGAATTGGTTTCGGTCATTTTCTGCTTAGAGAGTAAGCTAAGTTACTA<br>TTGTATTGGTTTATAAAGACTGTGGCTTTTAGTTGGTTTAGAGCAGTTCTCTCCTATCTTGT<br>TGGTTTGATAATAGTAACTGAATGAAGATCCTAGAGATTTTAAGTAATCACAGCTTTGATGT<br>TGTGTGAATGCAG
Δ2	GTTTGTGTGTTTCTCGTATCTGATAAATGTCAATCCTTTAGTAGAAGAAATTCATTGCTGGA<br>TTTGAATAGATTCAAGTTTTAAG(*TAGAAGAGGTCACACATTTCTTCAGAATTGCTGGATTT*<br>*GAGAACCTGAATTGAATTGGTTTCGGTCATTTTCTGCTTAGAGAGTAAGCTA*)AGTTACTA<br>TTGTATTGGTTTATAAAGACTGTGGCTTTTAGTTGGTTTAGAGCAGTTCTCTCCTATCTTGT<br>TGGTTTGATAATAGTAACTGAATGAAGATCCTAGAGATTTTAAGTAATCACAGCTTTGATGT<br>TGTGTGAATGCAG
Δ3	GTTTGTGTGTTTCTCGTATCTGATAAATGTCAATCCTTTAGTAGAAGAAATTCATTGCTGGA<br>TTTGAATAGATTCAAGTTTTAAGTAGAAGAGGTCACACATTTCTTCAGAATTGCTGGATTT<br>GAGAACCTGAAT(*TGAATTGGTTTCGGTCATTTTCTGCTTAGAGAGTAAGCTAAGTTACTA*<br>*TTGTATTGGTTTATAAAGACTGTGGCTTTTAGTTGGTTTAGA*)GCAGTTCTCTCCTATCTTGT<br>TGGTTTGATAATAGTAACTGAATGAAGATCCTAGAGATTTTAAGTAATCACAGCTTTGATGT<br>TGTGTGAATGCAG
Δ4	GTTTGTGTGTTTCTCGTATCTGATAAATGTCAATCCTTTAGTAGAAGAAATTCATTGCTGGA<br>TTTGAATAGATTCAAGTTTTAAGTAGAAGAGGTCACACATTTCTTCAGAATTGCTGGATTT<br>GAGAACCTGAATTGAATTGGTTTCGGTCATTTTCTGCTTAGAGAGTAAGCTAAGTTACTAT<br>TGTATTGGTTTATAAAGACTGTG(*GCTTTTAGTTGGTTTAGAGCAGTTCTCTCCTATCTTGTT*<br>*GGTTTGATAATAGTAACTGAATGAAGATCCTAGAGATTTTAAGTAATCACA*)GCTTTGATGT<br>TGTGTGAATGCAG
ΔNAC1	GTTTGTGTGTTTCTCGTATCTGATAAATGTCAATCCTT**cg**GTAGAAGAAATTCATTGCTGGA<br>TTTGAATAGATTCAAGTTTTAAGTAGAAGAGGTCACACATTTCTTCAGAATTGCTGGATTT<br>GAGAACCTGAATTGAATTGGTTTCGGTCATTTTCTGCTTAGAGAGTAAGCTAAGTTACTAT<br>TGTATTGGTTTATAAAGACTGTGGCTTTTAGTTGGTTTAGAGCAGTTCTCTCCTATCTTGTT<br>GGTTTGATAATAGTAACTGAATGAAGATCCTAGAGATTTTAAGTAATCACAGCTTTGATGTT<br>GTGTGAATGCAG
ΔNAC2	GTTTGTGTGTTTCTCGTATCTGATAAATGTCAATCCTTTAGTAGAAGAAATTCATTGCTGGA<br>TTTGAATAGATTCAAGTTTTAAGTAGAAGAGGTCACACATTTCTTCAGAATTGCTGGATTT<br>GAGAACCTGAATTGAATTGGTTTCGGTCATTTTCTG**tc**TAGAGAGTAAGCTAAGTTACTAT<br>TGTATTGGTTTATAAAGACTGTGGCTTTTAGTTGGTTTAGAGCAGTTCTCTCCTATCTTGTT<br>GGTTTGATAATAGTAACTGAATGAAGATCCTAGAGATTTTAAGTAATCACAGCTTTGATGTT<br>GTGTGAATGCAG
ΔNAC3	GTTTGTGTGTTTCTCGTATCTGATAAATGTCAATCCTTTAGTAGAAGAAATTCATTGCTGGA<br>TTTGAATAGATTCAAGTTTTAAGTAGAAGAGGTCACACATTTCTTCAGAATTGCTGGATTT<br>GAGAACCTGAATTGAATTGGTTTCGGTCATTTTCTGCTTAGAGAGTAAGCTAAGTTACTAT<br>TGTATTGGTTTATAAAGACTGTGGCTTTTAGTTGGTTTAGAGCAGTTCTCTCCTATCTTGTT

	GGTTTGATAATAGTAACTGAATGAAGATCCTAGAGATTTTA**ct**TAATCACAGCTTTGATGTT<br>GTGTGAATGCAG
ΔMYB1	GTTTGTGTGTTTCTCGTATCTGATAAATGTCAATCCTTTAGTAGAAGAAATTCATTGCTGGA<br>TTTGAATAGATTCAAGTTTTAAGTAGAAGAGGTCACACATTTCTTCAGAATTGCTGGATTT<br>GAGAACCTGAATTGAAT**ca**GTTTCGGTCATTTTCTGCTTAGAGAGTAAGCTAAGTTACTAT<br>TGTATTGGTTTATAAAGACTGTGGCTTTTAGTTGGTTTAGAGCAGTTCTCTCCTATCTTGTT<br>GGTTTGATAATAGTAACTGAATGAAGATCCTAGAGATTTTAAGTAATCACAGCTTTGATGTT<br>GTGTGAATGCAG
ΔMYB2	GTTTGTGTGTTTCTCGTATCTGATAAATGTCAATCCTTTAGTAGAAGAAATTCATTGCTGGA<br>TTTGAATAGATTCAAGTTTTAAGTAGAAGAGGTCACACATTTCTTCAGAATTGCTGGATTT<br>GAGAACCTGAATTGAATTGGTTTCGGTCATTTTCTGCTTAGAGAGTAAGCTAAGTTACTAT<br>TGTATTGGTTTATAAAGACTGTGGCTTTTAGTTGGTTTAGAGCAGTTCTCTCCTATCTTGTT<br>GGTTTGATAATAGTA**ct**TGAATGAAGATCCTAGAGATTTTAAGTAATCACAGCTTTGATGTT<br>GTGTGAATGCAG
ΔNACs	GTTTGTGTGTTTCTCGTATCTGATAAATGTCAATCCTT**cg**GTAGAAGAAATTCATTGCTGGA<br>TTTGAATAGATTCAAGTTTTAAGTAGAAGAGGTCACACATTTCTTCAGAATTGCTGGATTT<br>GAGAACCTGAATTGAATTGGTTTCGGTCATTTTCTG**tc**TAGAGAGTAAGCTAAGTTACTAT<br>TGTATTGGTTTATAAAGACTGTGGCTTTTAGTTGGTTTAGAGCAGTTCTCTCCTATCTTGTT<br>GGTTTGATAATAGTAACTGAATGAAGATCCTAGAGATTTTA**ct**TAATCACAGCTTTGATGTT<br>GTGTGAATGCAG
ΔMYBs	GTTTGTGTGTTTCTCGTATCTGATAAATGTCAATCCTTTAGTAGAAGAAATTCATTGCTGGA<br>TTTGAATAGATTCAAGTTTTAAGTAGAAGAGGTCACACATTTCTTCAGAATTGCTGGATTT<br>GAGAACCTGAATTGAAT**ca**GTTTCGGTCATTTTCTGCTTAGAGAGTAAGCTAAGTTACTAT<br>TGTATTGGTTTATAAAGACTGTGGCTTTTAGTTGGTTTAGAGCAGTTCTCTCCTATCTTGTT<br>GGTTTGATAATAGTA**ct**TGAATGAAGATCCTAGAGATTTTAAGTAATCACAGCTTTGATGTT<br>GTGTGAATGCAG
ΔNACs<br>&MYBs	GTTTGTGTGTTTCTCGTATCTGATAAATGTCAATCCTT**cg**GTAGAAGAAATTCATTGCTGGA<br>TTTGAATAGATTCAAGTTTTAAGTAGAAGAGGTCACACATTTCTTCAGAATTGCTGGATTT<br>GAGAACCTGAATTGAAT**ca**GTTTCGGTCATTTTCTG**tc**TAGAGAGTAAGCTAAGTTACTATT<br>GTATTGGTTTATAAAGACTGTGGCTTTTAGTTGGTTTAGAGCAGTTCTCTCCTATCTTGTTG<br>GTTTGATAATAGTA**ct**TGAATGAAGATCCTAGAGATTTTA**ct**TAATCACAGCTTTGATGTTGT<br>GTGAATGCAG

Supplemental table 3. Identity matrix for the first intron downstream of the translational start site in diverse flowering plants, related to Figure 5.

Gene ID	Organism	Percentage identity with AtARF7 (Exon1>Intron>Exon2)	Percentage identity with AtARF7 (Intron)	First exon length	Intron length	Second Exon length
AT5G20730.3	*A.thaliana*	--	--	46	320	113
BnaA10g14760D	*B. napus*	74%	63%	46	292	113
Bo2g023110	*B. oleracea*	66%	51.10%	46	247	113
MTR_4g124900	*M. truncatula*	28.40%	20.50%	43	943	113
EJB05_45841	*E. curvula*	38.50%	34.20%	40	253	113
OsI_021248	*O. sativa*	39.80%	23.70%	46	248	113
Zm00001eb373970	*Z. mays*	41.70%	30.70%	46	261	113

Percentage identity with AtARF7 in column three refers to a total sequence identity from the start of exon 1 until the end of exon 2.

Supplemental table 4. Putative binding sites within the first intron downstream of the translational start site in diverse flowering plants, related to Figure S5. Transcription factors were predicted for intronic sequence using the PlantRegMap TF database.

**TF Family**	*B. napus*	*A. thaliana*	*B. oleracea*	*M. truncatula*	*E. curvula*	*O. sativa*	*Z. mays*
G2-like	1	1	0	3	0	0	0
NAC	2	3	1	0	1	0	1
WRKY	1	0	0	0	0	0	0
MYB/MYB related	1	2	1	0	0	0	0
EIL	1	0	0	1	1	0	0
MIKC_MADS	2	0	0	2	2	0	1
AP2	1	0	0	2	0	0	0
Dof	1	0	1	2	0	0	0
C2H2	2	0	0	5	1	1	2
E2F/DP	1	0	0	0	0	0	0
TCP	2	0	0	0	0	0	1
ARR-B	0	1	0	0	0	0	0
CPP	0	1	0	0	0	0	0
GRAS	0	1	0	4	0	0	1
HSF	0	1	0	0	0	0	1
WOX	0	1	0	1	0	0	0
BBR-BPC	0	0	0	1	1	0	0
GATA	0	0	0	1	0	0	1
HD-ZIP	0	0	0	1	1	0	1
SRS	0	0	0	1	0	0	0
ZF-HD	0	0	0	1	0	0	0
ERF	0	0	0	0	0	1	0
bZIP	0	0	0	0	0	0	1
TALE	0	0	0	0	0	0	1

Supplemental Table 5. List of Greengate modules used in this study, relating to STAR methods.

Module	Name	Information
Entry A	pGGA-ARF7proS	
	pGGA-ARF7UTRINT	Gibson
	pGGA-ARF7INTUTR	Gibson
Entry B	pGGB003-B dummy	GreenGate System
	pGGB-EX1	
	pGGB-EX1-2	
	pGGB-EX1-2 (d1)	Gibson
	pGGB-EX1-2 (d2)	Gibson
	pGGB-EX1-2 (d3)	Gibson
	pGGB-EX1-2 (d4)	Gibson
	pGGB-EX1-2 (Δ1stNAC)	
	pGGB-EX1-2 (Δ2ndNAC)	
	pGGB-EX1-2 (Δ3rdNAC)	
	pGGB-EX1-2 (Δ3*NAC)	Synthesised from EurofinsGenomics
	pGGB-EX1-2 (Δ2*MYB)	Synthesised from EurofinsGenomics
Entry C	pGGC012-GFP	GreenGate System
Entry D	pGGD003-D dummy	GreenGate System
Entry E	pGGE001-RBCS	GreenGate System
Entry F	pGGF005-Hygromycin B	GreenGate System
	pGGZ003-DEAL	By Britta Kuempers

Supplemental Table 6. All primers used in this study, relating to STAR methods

	Primer Name	Sequence	
1	pARF7sF	AACAGGTCTCTACCTaagagATGTCGCAAACCAGC	
2	pARF7sR	AACAGGTCTCGTGTTgatcactcaactttactttctctgaa	
3	pARF7pro-seqF	caccatacggacacacaacc	For sequencing
4	pARF7pro-seqR	ccgacatattcactagcaaactgg	For sequencing
5	pARF7Ex1-2f	gcttggtctcaaacaccATGAAAGCTCCTTCATCAAATGGAG	
6	pARF7ex1-2r	attcggtctcaagccCCTTTCTCctgcattcacaca	
7	pARF7Ex1f	gcttggtctcaaacaccATGAAAGCTCCTTCATCAAATGGAG	
8	pARF7Ex1r	attcggtctcaagcCTTCAACAGGATTAGGAGAAACTCC	
9	pGibARF7prosf	ttgtgtgaatgcagGAGAAAttatttattgggtttattcttcagaga	
10	pGibARF7prosr	agaaacacacaaacCTTCAAtctgaatctgagcttatacaaag	
11	pGibARF7INTf	tgtataagctcagattcagaTTGAAGgtttgtgtgtttctc	
12	pGibARF7INTr	agaataaacccaataaataaTTTCTCctgcattcacacaa	
13	pARF7proGib-seqF	cagagataaggaaaaagaaaacac	For sequencing
14	NAC1F	GTCAATCCTTcgGTAGAAGAAATTCATTG	
15	NAC1R	ATTTATCAGATACGAGAAACAC	
16	NAC f	TCATTTTCTGtcTAGAGAGTAAGCTAAG	
17	NAC r	CCGAAACCAATTCAATTC	
18	NAC2F	AGAGATTTTActTAATCACAGCTTTGATGTTGTGTG	
19	NAC2R	AGGATCTTCATTCAGTTAC	
20	D1F	TTTCTTCAGAATTGCTGG	
21	D1R	AACACACAAACCTTCAAC	
22	D2F	AGTTACTATTGTATTGGTTTATAAAG	
23	D2R	CTTAAAACTTGAATCTATTCAAATC	

24	D3F	GCAGTTCTCTCCTATCTTG	
25	D3R	ATTCAGGTTCTCAAATCC	
26	D4F	GCTTTGATGTTGTGTGAATG	
27	D4R	CACAGTCTTTATAAACCAATAC	
28	B-dummyF	GTATTCAGTCGACTGGTACC	For sequencing
29	B-dummyR	GGTACCAGTCGACTGAATAC	For sequencing
30	GFP-R	TGCAGATGAACTTCAGGGTC	For sequencing
31	D-dummyF	CAGGTGGATCCTAGATAACC	For sequencing
32	D-dummyR	GCAGGGTACCAATTTACAGG	For sequencing
33	RBCS-F	CCTTGTCAGATTCTAATCATTGC	For sequencing
34	HygR-R	AGGTCACGAGAAAGCTAAGG	For sequencing
35	EntryConF	gtggaattgtgagcggataac	For sequencing
36	EntryConR	GTTTTCCCAGTCACGACGTT	For sequencing
59	ARF7-TRANSF	agttccccatttctgattaacg	For RT-PCR
60	ARF7-TRANSR	TGAACTTGTGGCCGTTTAC	For RT-PCR
61	GFP-F	TTCAAGGACGACGGCAACTA	For qRT-PCR
62	GFP-R	TCAGCTCGATGCGGTTCA	For qRT-PCR
